# ATOH1, TFAP2B, and CEACAM6 as Immunohistochemical Markers to Distinguish Merkel Cell Carcinoma and Small Cell Lung Cancer

**DOI:** 10.3390/cancers16040788

**Published:** 2024-02-15

**Authors:** Serena M. Vilasi, Jannett Nguyen, Catherine J. Wang, Lingling Miao, Kenneth Daily, Mary Eid, Joon Seon Song, Hong Jiang, Kris Ylaya, Klaus J. Busam, Maria R. Gaiser, Stephen M. Hewitt, Isaac Brownell

**Affiliations:** 1 Dermatology Branch, National Institute of Arthritis and Musculoskeletal and Skin Diseases, National Institutes of Health, Bethesda, MD 20892, USA; 2 Laboratory of Pathology, Center for Cancer Research, National Cancer Institute, National Institutes of Health, Bethesda, MD 20892, USA; 3 Dermatopathology Service, Memorial Sloan Kettering Cancer Center, New York City, NY 10065, USA; 4 Department of Dermatology, University of Heidelberg, 69120 Heidelberg, Germany

**Keywords:** Merkel cell carcinoma, small cell lung cancer, neuroendocrine tumor, ATOH1, TFAP2B, CEACAM6

## Abstract

**Simple Summary:**

Merkel cell carcinoma (MCC) and small cell lung cancer (SCLC) are both neuroendocrine cancers that can resemble each other under the microscope. Immunostaining with diagnostic markers such as cytokeratin 20 (CK20) and thyroid transcription factor 1 (TTF-1) can help differentiate these two cancers, but their sensitivity and specificity are limited. We compared the gene expression of MCC and SCLC tumors to identify highly differentially expressed genes for potential use as diagnostic markers. Two candidate markers for MCC, atonal BHLH transcription factor 1 (ATOH1) and transcription factor AP-2β (TFAP2B), and one candidate marker for SCLC, carcinoembryonic antigen cell adhesion molecule 6 (CEACAM6), were tested using immunostaining. Combined use of CEACAM6 with TTF-1 increased SCLC diagnostic sensitivity to 93% and specificity to 98%. A panel of CK20, ATOH1, and TFAP2B was 100% sensitive and specific for MCC, suggesting the potential utility of these new markers in differentiating MCC and SCLC.

**Abstract:**

Merkel cell carcinoma (MCC) and small cell lung cancer (SCLC) can be histologically similar. Immunohistochemistry (IHC) for cytokeratin 20 (CK20) and thyroid transcription factor 1 (TTF-1) are commonly used to differentiate MCC from SCLC; however, these markers have limited sensitivity and specificity. To identify new diagnostic markers, we performed differential gene expression analysis on transcriptome data from MCC and SCLC tumors. Candidate markers included atonal BHLH transcription factor 1 (ATOH1) and transcription factor AP-2β (TFAP2B) for MCC, as well as carcinoembryonic antigen cell adhesion molecule 6 (CEACAM6) for SCLC. Immunostaining for CK20, TTF-1, and new candidate markers was performed on 43 MCC and 59 SCLC samples. All three MCC markers were sensitive and specific, with CK20 and ATOH1 staining 43/43 (100%) MCC and 0/59 (0%) SCLC cases and TFAP2B staining 40/43 (93%) MCC and 0/59 (0%) SCLC cases. TTF-1 stained 47/59 (80%) SCLC and 1/43 (2%) MCC cases. CEACAM6 stained 49/59 (83%) SCLC and 0/43 (0%) MCC cases. Combining CEACAM6 and TTF-1 increased SCLC detection sensitivity to 93% and specificity to 98%. These data suggest that ATOH1, TFAP2B, and CEACAM6 should be explored as markers to differentiate MCC and SCLC.

## 1. Introduction

Merkel cell carcinoma (MCC) is a rare and aggressive neuroendocrine skin cancer [1]. Histologically, MCC is characterized by collections of monotonous small round blue cells, typically in the dermis and subcutaneous tissues, with high nuclear-to-cytoplasmic ratios and pale or dense hyperchromatic nuclei with finely stippled chromatin [2]. Metastatic lesions from other neuroendocrine tumors, such as small cell lung cancer (SCLC) or gastrointestinal carcinoid tumors, have morphologic and cytologic findings similar to MCC [2]. Thus, histology alone is inadequate for distinguishing between them and immunohistochemistry (IHC) is required for diagnosis [2]. Compared to gastrointestinal neuroendocrine tumors, SCLC is highly aggressive and more likely to metastasize [3]. In 20–60% of cases of lung cancer with cutaneous metastases, skin lesions present prior to or synchronously with the diagnosis of the primary lung tumor [3].

Considering their potential similarities, it is important to distinguish MCC from cutaneous metastases of SCLC to ensure accurate pathologic diagnosis and guide appropriate management. Currently, cytokeratin 20 (CK20) and thyroid transcription factor-1 (TTF-1) are the most widely used markers for differentiating between MCC and SCLC, with CK20 expression favoring MCC and TTF-1 expression favoring SCLC [2]. A meta-analysis of published studies reporting on IHC staining of these markers in MCC and SCLC demonstrated that CK20 has an aggregate sensitivity of 86% and specificity of 89% for MCC, and TTF-1 has a sensitivity of 85% and specificity of 97% for SCLC (Table 1). Considering the limitations of these markers, we sought to systematically identify new IHC markers to distinguish MCC and SCLC.

## 2. Materials and Methods

### 2.1. Case Selection

This study analyzed deidentified tumor samples in accordance with Institutional Review Board approved protocols. MCC cases were obtained from the Memorial Sloan Kettering Cancer Center (MSKCC) (New York, NY, USA), Heidelberg University (Heidelberg, Germany), and University of Ulsan College of Medicine, Asan Medical Center (Seoul, Republic of Korea). SCLC cases were obtained from the Memorial Sloan Kettering Cancer Center (MSKCC) (New York, NY, USA) and US Biomax, Inc. (Derwood, MD, USA). The diagnosis of each case was verified by histological review prior to inclusion in the study.

### 2.2. Differential Gene Expression Analysis

Fresh frozen tumor specimens were obtained from the MSKCC tumor bank. Total RNA from 23 MCC and 9 SCLC tumor samples was extracted and hybridized to Human Genome U122A 2.0 Array GeneChips (Affymetrix, Santa Clara, CA) as previously reported [29]. Microarray expression data were normalized, filtered, and analyzed in R (version 2.13.0). Using the Limma (version 3.21.15) package within the R environment [30] for analysis, a probe set was considered highly differentially expressed if the absolute log2 fold change was greater than 3 and the adjusted *p*-value was less than 1 × 10^−10^. Candidate genes were selected based on differential mRNA expression and the availability of validated antibodies for IHC.

### 2.3. SCLC Subtype Analysis

SCLC samples were subtyped based on the normalized expression of neuroendocrine markers and the characteristic subtype transcription factors *ASCL1*, *NEUROD1*, *POU2F3*, and *YAP1* [31,32]. When subtype classification was not clear based on the above markers, expression levels of *MYC* and *MYCL* along with full transcriptome phylogenetic relationships among the tumors were used to best estimate the sample subtypes.

### 2.4. Immunohistochemistry

IHC was performed on formalin-fixed, paraffin-embedded tissue sections and tissue microarrays. In total, 43 MCC cases and 59 SCLC cases were stained with atonal BHLH transcription factor 1 (ATOH1 rabbit polyclonal antibody, ab105497, 1:250; Abcam, Cambridge, MA, USA) and transcription factor AP-2β (mouse monoclonal antibody TFAP2B, sc-390281, 1:1500; Santa Cruz Biotechnology Inc., Santa Cruz, CA, USA) antibodies using a Dako (Santa Clara, CA, USA) Autostainer Universal Staining System. Antigen retrieval consisted of heat-induced epitope retrieval in a pressure cooker in a pH 6 target retrieval solution (Dako) for 40 min, up to 125 °C and 20 psi. Slides were then treated with a 3% hydrogen peroxide enzyme block (Dako) for 5 min, a serum-free protein block (Dako) for 15 min, a primary antibody for 60 min at room temperature, and an Envision+ goat anti-rabbit (Dako) or Envision+ goat anti-mouse (Dako) secondary antibody for 30 min at room temperature. The bound antibody was detected using a DAB+ substrate kit (Dako). Cases were also stained with clinically validated antibodies for CK20 (KS20.8, prediluted; Dako), TTF-1 (SP141, prediluted; Roche, Tuscon, AZ, USA), and carcinoembryonic antigen polyclonal antibody (CEA-P, prediluted; Dako) on a Ventana Benchmark Ultra (Tucson, AZ, USA) automated slide staining system according to the manufacturer’s instructions. Hematoxylin was used for counterstaining.

### 2.5. Analysis of Expression

The samples were assessed independently by two pathologists from the NIH Laboratory of Pathology who were blinded to the tissue source. Immunoreactivity was assessed using a semiquantitative system combining staining intensity and proportion of cells stained. A score of 0 indicated no staining or <1% cells stained, 1 indicated weak focal or weak diffuse staining, 2 indicated moderate focal, moderate diffuse, or strong focal staining, and 3 indicated strong diffuse staining. For sensitivity and specificity analyses, a score of 0 was considered negative and scores of 1, 2, or 3 were considered positive. Samples with scoring discrepancies were discussed until an agreement was reached. McNemar’s test was used to compare the sensitivities and specificities of antibody panels, and *p*-values less than 0.05 were considered significant.

## 3. Results

### 3.1. Differential Gene Expression in MCC and SCLC

**Figure 1:**
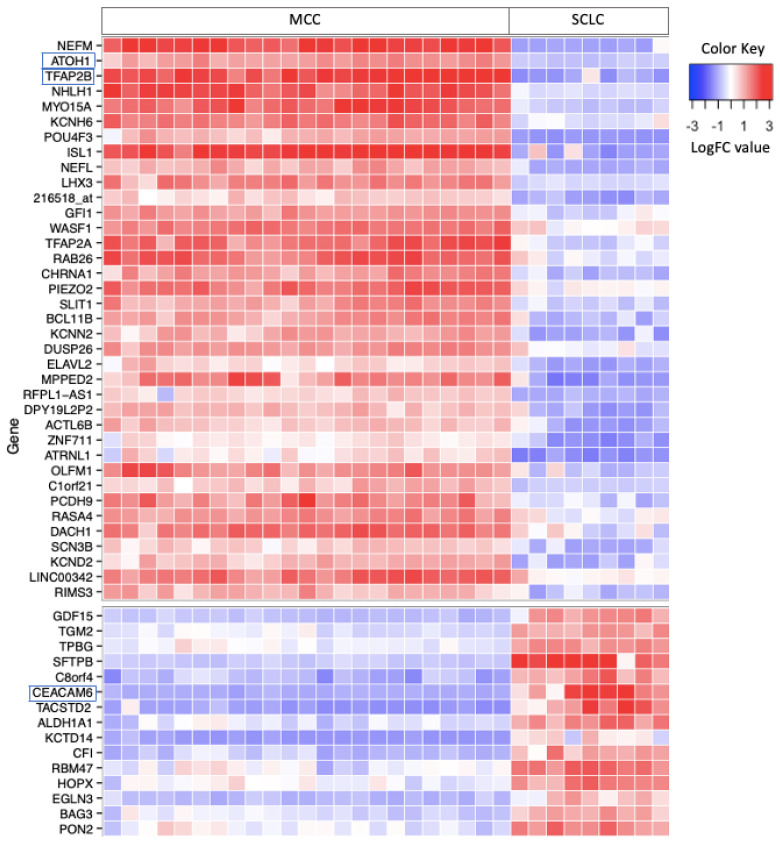
Heat map of highly differentially expressed protein-coding genes in MCC and SCLC tumors. Differentially expressed genes (*n* = 48) have an absolute log2 fold change (LogFC) greater than 3 and an adjusted p-value less than 1 × 10^−10^. Candidate genes (annotated in boxes) ATOH1 and TFAP2B were selected as IHC markers for MCC and CEACAM6 was selected for SCLC. MCC, Merkel cell carcinoma. SCLC, small cell lung cancer.

Microarray transcriptome analysis identified 48 highly differentially expressed protein-coding genes in MCC and SCLC tumors, which served as candidate markers for IHC (Figure 1). Interestingly, genes for the standard markers CK20 and TTF-1 were not among the top 48 differentially expressed transcripts. Neurofilament proteins have previously been described as a marker for MCC, and the medium and light chain genes (*NEFM* and *NEFL*) demonstrated high differential expression in MCC. From this list of candidate markers, we tested ATOH1 and TFAP2B as potential new IHC markers for MCC and carcinoembryonic antigen cell adhesion molecule 6 (CEACAM6) as a novel marker for SCLC.

### 3.2. SCLC Subtype Analysis

**Figure 2:**
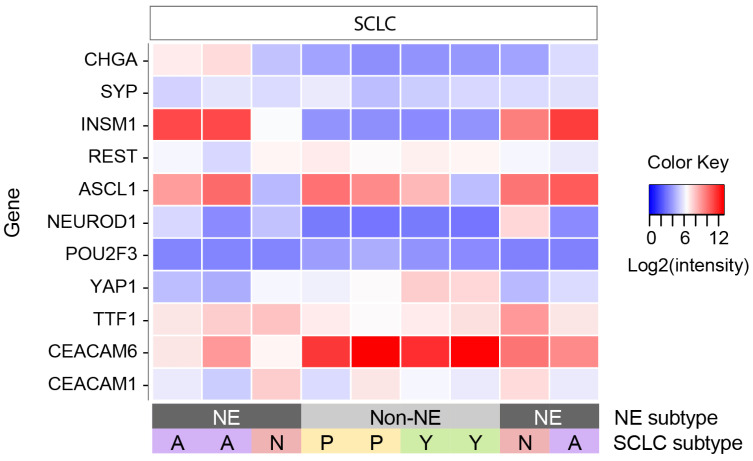
SCLC subtypes based on microarray gene expression data. Heat map of genes used to subtype SCLC samples and candidate marker genes. Samples are in the same order as in Figure 1. SCLC, small cell lung cancer. NE, neuroendocrine.

SCLC tumors have distinct molecular subtypes [31]. Some SCLC tumors have higher expression of neuroendocrine (NE) marker genes such as *CHGA*, *SYP*, and *INSM1,* whereas non-NE tumors express higher levels of *REST* [32]. The NE tumors can be further divided into SCLC-A and SCLC-N subtypes based on the expression of the transcription factors *ASCL1* and *NEUROD1*, respectively. The non-NE tumors can be subtyped into SCLC-P and SCLC-Y based on the relative expression of *POU2F3* and *YAP1*. Although the subtyping of SCLC tumors using microarray expression data has not been validated, we were able to subtype the SCLC samples used for the differential gene expression analysis (Figure 2). Notably, our cohort included tumors of all four SCLC subtypes. Although the non-NE tumors appeared to have the highest levels of the candidate marker gene, *CEACAM6* was expressed by tumors of all SCLC subtypes.

### 3.3. Immunohistochemical Staining

**Figure 3:**
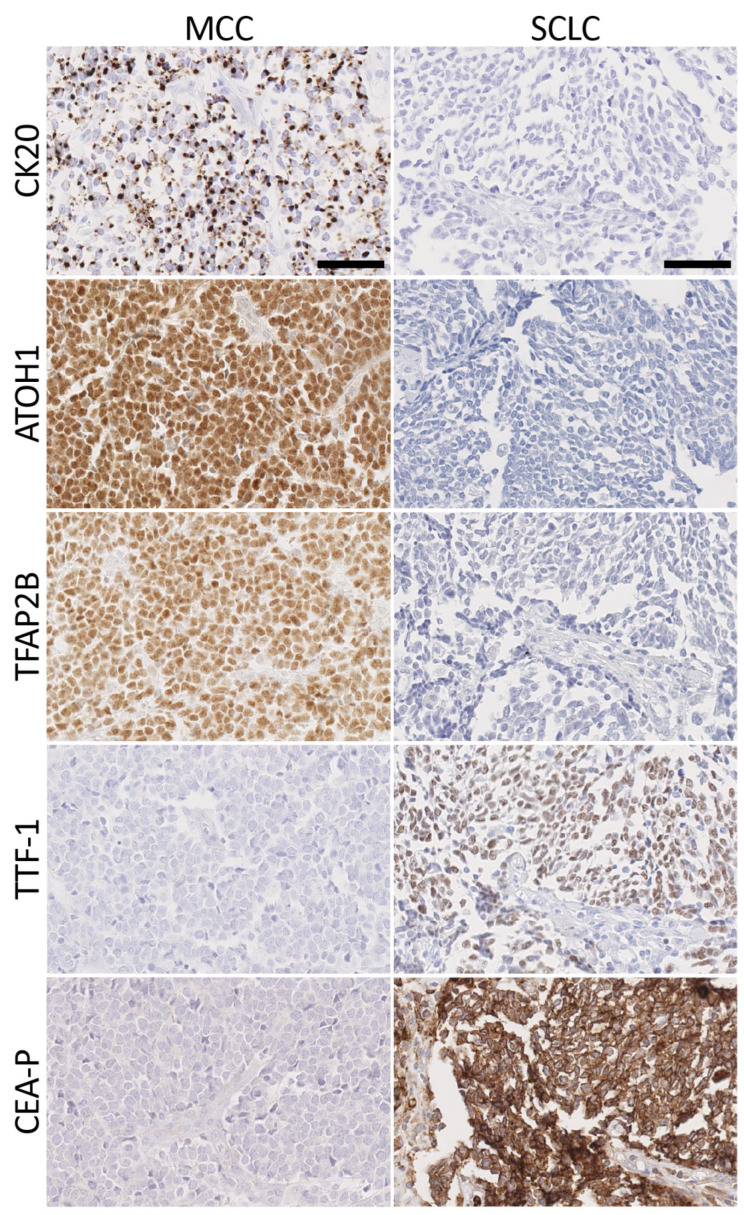
Immunohistochemical staining for standard and new markers for MCC and SCLC. MCC markers (CK20, ATOH1, and TFAP2B) and SCLC markers (TTF-1 and CEA-P) were used to stain FFPE tissue sections. Images were taken on a NanoZoomer Digital slide scanner at 200× magnification. MCC, Merkel cell carcinoma. SCLC, small cell lung cancer. Scale bars = 100 μm.

IHC staining of tissue sections from MCC (*n* = 43) and SCLC (*n* = 59) tumors was performed with CK20, ATOH1, TFAP2B, TTF-1, and CEA-P antibodies (Table 2 and Figure 3). CEA-P is a rabbit polyclonal antibody that recognizes CEACAM1 and CEACAM6.

### 3.4. Merkel Cell Carcinoma Markers

CK20 was expressed in a paranuclear dot or cytoplasmic pattern or both in 100% (43/43) of MCC cases and 93.0% (40/43) had moderate or strong staining. The extent of CK20 staining was generally diffuse. All 59 cases of SCLC were CK20-negative. Thus, in our cohort of cases, CK20 was 100% sensitive and 100% specific for distinguishing MCC from SCLC. 

ATOH1 stained 100% (43/43) of MCC cases in a nuclear pattern, and 90.7% (39/43) had moderate or strong staining. The extent of ATOH1 staining was generally diffuse. All 59 cases of SCLC were ATOH1-negative. As such, ATOH1 was 100% sensitive and 100% specific for MCC.

TFAP2B was expressed in a nuclear pattern in 93.0% (40/43) of MCC, and 74.4% (32/43) had moderate or strong staining. The extent of TFAP2B staining was generally diffuse. All 59 SCLC tumors were TFAP2B-negative. Overall, TFAP2B was 93% sensitive and 100% specific for MCC.

### 3.5. Small Cell Lung Cancer Markers

TTF-1 was expressed in a nuclear pattern in 79.7% (47/59) of SCLC tumors and 74.6% (44/59) had moderate or strong staining. One MCC sample (1/43) had a diffuse nuclear expression of TTF-1 with moderate intensity. In all, TTF-1 was 80% sensitive and 98% specific for distinguishing SCLC from MCC.

The CEA-P antibody used in this study binds both CEACAM1 and CEACAM6 proteins. As shown in Figure 1, CEACAM6 was among the most highly differentially expressed genes in SCLC. While not among the top differentially expressed genes, CEACAM1 was also expressed at higher levels in SCLC relative to MCC (see Figure 2). The CEA-P antibody stained 83.1% (49/59) of SCLC samples in a cytoplasmic and membranous pattern, and 64.4% (38/59) had moderate or strong staining. All 43 MCC tumors were negative. Overall, CEA-P was 83% sensitive and 100% specific for SCLC.

### 3.6. Comparison of Antibody Panels

Combining IHC markers into panels can improve diagnostic sensitivity and specificity. We interpreted an antibody panel as positive if staining was positive for at least one marker in the panel. As CK20 and ATOH1 were 100% sensitive and specific for MCC in our cohort of tumors, all panel combinations that included either marker were also 100% sensitive and specific (Table 2). Although TFAP2B had a lower sensitivity (93%) for MCC, its diagnostic performance was not significantly different compared to CK20 or ATOH1 (*p*-value > 0.05). 

For diagnosing SCLC, a panel combining CEA-P and TTF-1 increased sensitivity to 93%, which was significantly higher than either TTF-1 (80% sensitive) or CEA-P (83% sensitive) (*p*-value < 0.05). The lower specificity of this dual panel (98% specific) compared to CEA-P alone (100% specific) was not statistically significant (*p*-value > 0.05).

## 4. Discussion

The incidence of MCC has increased in recent decades due to advances in diagnostic IHC markers, an aging population, and increased use of immunosuppressive therapies [33]. Concomitantly, there has been interest in identifying IHC markers like CK20 and TTF-1 that can help differentiate MCC from SCLC. For example, achaete-scute homolog 1 (ASCL1) was proposed as a new SCLC marker that showed 83% sensitivity and 100% specificity in differentiating SCLC from MCC [16]. In another study, anaplastic lymphoma kinase (ALK) was proposed as a new MCC marker with one antibody clone (D5F3) demonstrating 94% sensitivity and 92% specificity for distinguishing MCC from SCLC [25]. 

In this study, we used differential gene expression analysis of global transcriptome data to identify candidate IHC markers that distinguish MCC and SCLC. For MCC, we describe two new IHC markers, ATOH1 and TFAP2B, both of which have high sensitivity and specificity and could be useful adjuncts to CK20. For SCLC, we discovered CEA-P as a new IHC marker with a diagnostic sensitivity and specificity comparable to TTF-1 and that the combined use of both markers significantly increases the sensitivity for SCLC. 

CK20 is an established and useful diagnostic marker for MCC. Although all MCC tumors in this study expressed CK20, only 86% (515/599) of published MCC cases were CK20-positive (Table 1). In addition, 11% (52/477) of SCLC samples were CK20-positive, hampering the specificity of this marker (Table 1). Thus, additional markers like ATOH1 and TFAP2B could be useful adjuncts for diagnosing MCC. 

In contrast to CK20, which is expressed in the cytoplasm, ATOH1 and TFAP2B are transcription factors expressed in the nucleus. ATOH1 is a transcription factor required for normal Merkel cell development [34]. In MCC tumors, high *ATOH1* expression assessed by IHC correlates with tumor relapse, which may position ATOH1 as a marker with both diagnostic and prognostic relevance [35].

Transcriptional regulation by *TFAP2B* is involved in many functions including the regulation of cell division, apoptosis, and the differentiation of neural crest cells [36]. Although the role of *TFAP2B* in MCC has not been described, the TFAP2 family is known to play multiple roles in cancer development [37].

Neurofilament (*NF*) component genes were among the highly differentially expressed genes in our analysis. NF has been reported as an MCC marker in a number of studies [8,12,24]. Based on the aggregate staining of cases reported in these studies, NF is 78% sensitive and 98% specific for distinguishing MCC from SCLC. However, our attempts to reliably detect NF staining in MCC tumors using clinically validated NF antibodies were unsuccessful. 

Among published SCLC cases, TTF-1 IHC was positive in 85% (314/371) of tumors (Table 1), and we found a similar sensitivity of 80% in our SCLC cases with a specificity of 98%. We describe CECAM6 detection with CEA-P IHC as a new SCLC marker with 83% sensitivity and 100% specificity for distinguishing SCLC from MCC. A panel combining both TTF-1 and CEA-P significantly increases sensitivity to 93%, suggesting that CEA-P staining compensates for cases in which TTF-1 is negative. Unlike TTF-1, which is expressed in the nucleus, the CEACAM proteins stained by the CEA-P antibody are cytoplasmic. The role of CEACAM proteins in SCLC is unknown, but high surface expression of CEACAM1 is associated with microvessel density, distant metastases, and shorter median overall survival in non-small cell lung cancer, whereas high surface expression of CEACAM6 is associated with poor tumor differentiation in colon adenocarcinoma [38,39].

Of note, we report one case of MCC with diffuse TTF-1 staining, a rare phenomenon in MCC with few documented cases [40,41,42,43,44,45]. TTF-1 expression has been shown in combined tumors that have both MCC and non-MCC (e.g., basal, squamous, follicular) components [46,47,48]. These tumors tend to be MCPyV negative and have high mutational burdens [48,49], and it is speculated that TTF-1 expression in these samples is related to chronic UV exposure [50]. 

Our study was limited by several factors. In searching for IHC diagnostic markers, we only pursued candidate genes whose products had antibodies that worked well on clinical tumor specimens. This approach ignored the biological relevance of the markers and excluded many differentially expressed genes. In addition, we did not consider other molecular markers that may be effective in differentiating MCC from SCLC such as genomic structural variants, DNA mutational signatures, epigenetic profiling, tumor exosomes, fusion transcripts, and circular RNA profiling. Our study was also limited by the small number of samples used for gene expression analysis, which could impact the generalizability of the findings and the robustness of the identified markers. Moreover, the small sample size precluded a meaningful comparison of tumor biology between MCC and SCLC or among the tumor subtypes. We tested the IHC markers in a larger set of tumors that were nonetheless limited by a lack of CK20-negative MCC cases and CK20-positive SCLC cases, which impaired our ability to compare the sensitivity and specificity of MCC markers. Nonetheless, by using an unbiased transcriptomic approach, we were able to identify new IHC markers for distinguishing MCC and SCLC. Future larger studies will be needed to validate our findings and better estimate the sensitivity and specificity of these markers, both alone and in combination with other diagnostic markers. Additional studies will also be needed to investigate whether these markers can help distinguish MCC from other types of metastatic neuroendocrine tumors, if they change with disease progression, or if they have potential utility in disease surveillance. 

## 5. Conclusions

The histological similarities between MCC and SCLC necessitate the use of IHC markers to make accurate pathological diagnoses. Meta-analysis of published studies demonstrated the most utilized diagnostic markers for this purpose, CK20 and TTF-1, which have limited specificity and sensitivity. In cases where these markers are insufficient for a clear diagnosis, using additional IHC markers such as ATOH1 and TFAP2B for MCC and CEA-P for SCLC may increase diagnostic sensitivity and specificity.

## Figures and Tables

**Table 1:** Meta-analysis of published studies reporting on CK20 and TTF-1 immunostaining in Merkel cell carcinoma and small cell lung cancer.

	MCC, % (+/*n*)		SCLC, % (+/*n*)	
Source	CK20+	TTF-1+	CK20+	TTF-1+
Byrd-Gloster et al. [4]	76% (16/21)	0% (0/21)	3% (1/36)	97% (35/36)
Cheuk et al. [5]	100% (23/23)	0% (0/23)	0% (0/52)	83% (43/52)
Hanly et al. [6]	95% (20/21)	0% (0/21)	33% (11/33)	85% (28/33)
Leech et al. [7]	91% (10/11)	0% (0/11)	0% (0/10)	100% (10/10)
Metz et al. [8]	100% (6/6)		0% (0/22)	
Ordonez [9]	76% (16/21)	0% (0/18)	4% (1/28)	96% (27/28)
Schmidt et al. [8], [10]	77% (43/56)		0% (0/18)	
Yang et al. [11]	86% (19/22)	0% (0/22)	0% (0/9)	100% (9/9)
Bobos et al. [12]	100% (13/13)	0% (0/13)	8% (1/13)	85% (11/13)
Fukuhara et al. [13]	75% (15/20)		0% (0/4)	
Sidiropoulos et al. [14]	88% (35/40)	3% (1/40)	0% (0/30)	77% (23/30)
Kolhe et al. [15]	94% (15/16)	0% (0/10)		100% (2/2)
Ralston et al. [16]		3% (1/30)		73% (43/59)
Moll et al. [17]	100% (15/15)		20% (3/15)	
Chan et al. [18]	97% (32/33)		3% (1/37)	
Chu et al. [19]	78% (7/9)		0% (0/7)	
Nicholson et al. [20]	67% (18/27)		0% (0/5)	
Kaufmann et al. [21]	86% (24/28)	0% (0/16)	0% (0/5)	81% (30/37)
Kervarrec et al. [22]	86% (12/14)	4% (1/28)	0% (0/7)	100% (5/5)
Rajagopalan et al. [23]	95% (37/39)		14% (4/28)	
Stanoszek et al. [24]	76% (37/49)		41% (25/61)	
Filtenborg-Barnkob et al. [25]	88% 28/32	22% (7/32)	33% (4/12)	100% (12/12)
Gandhi et al. [26]		0% (0/23)		56% (9/16)
Busam et al. [27]	89% (32/36)		0% (0/16)	
Cho et al. [28]	89% (42/47)	4% (2/47)	3% (1/29)	93% (27/29)
Cumulative Totals	86% (515/599)	3% (12/355)	11% (52/477)	85% (314/371)

MCC, Merkel cell carcinoma. SCLC, small cell lung cancer. CK20, cytokeratin 20. TTF-1, thyroid transcription factor 1.

**Table 2:** Sensitivities and specificities of individual IHC markers and combination panels for differentiating Merkel cell carcinoma and small cell lung cancer.

	MCC Markers, +/*n*						SCLC markers, +/*n*		
	Individual			Combination			Individual		Combination
	CK20	ATOH1	TFAP2B	CK20, ATOH1	CK20, TFAP2B	CK20, ATOH1, TFAP2B	TTF-1	CEA-P	TTF-1, CEA-P
MCC (*n* = 43)	43/43	43/43	40/43	43/43	43/43	43/43	1/43	0/43	1/43
SCLC (*n* = 59)	0/59	0/59	0/59	0/59	0/59	0/59	47/59	49/59	55/59
Sensitivity, %	100	100	93	100	100	100	80	83	93 *
Specificity, %	100	100	100	100	100	100	98	100	98 *

CK20, ATOH1, and TFAP2B were assessed as MCC markers. TTF-1 and CEA-P were assessed as SCLC markers. Combinations were considered positive if any marker in the panel was positive. * *p*-value < 0.05, when compared to either marker alone, by McNemar’s test. MCC, Merkel cell carcinoma. SCLC, small cell lung cancer. CK20, cytokeratin 20. TTF-1, thyroid transcription factor 1. ATOH1, atonal BHLH transcription factor 1. TFAP2B, transcription factor AP-2β.

## Data Availability

All microarray data for MCC and SCLC tumor samples have been deposited in the National Center for Biotechnology Information (NCBI) Gene Expression Omnibus and are accessible through Gene Expression Omnibus Series accession number GSE50451.
